# TPX2 Enhanced the Activation of the HGF/ETS-1 Pathway and Increased the Invasion of Endocrine-Independent Prostate Carcinoma Cells

**DOI:** 10.3389/fonc.2021.618540

**Published:** 2021-05-28

**Authors:** Hongqing Zhou, Mingsheng Liu, Tao Shao, Pingbo Xie, Shaojie Zhu, Wei Wang, Qiong Miao, Jiaxi Peng, Peng Zhang

**Affiliations:** ^1^ The Second Ward of Urology, Qujing Affiliated Hospital of Kunming Medical University, Qujing, China; ^2^ Department of Urology, Chinese People’s Liberation Army (PLA) General Hospital/Chinese PLA Medical Academy, Beijing, China

**Keywords:** the targeting protein for Xklp2, prostate carcinoma, E26 transformation specific sequence 1, metastasis, endocrine-independent

## Abstract

The prognosis for endocrine-independent prostate carcinoma is still poor due to its highly metastatic feature. In the present work, TPX2 (the targeting protein for Xklp2), which is known as a micro-tubulin interacted protein, was identified as a novel coactivator of ETS-1, a transcription factor that plays a central role in mediating the metastasis of human malignancies. TPX2 enhanced the transcription factor activation of ETS-1 and increased the expression of ETS-1’s downstream metastasis-related genes, such as *mmp3* or *mmp9*, induced by HGF (hepatocyte growth factor), a typical agonist of the HGF/c-MET/ETS-1 pathway. The protein-interaction between TPX2 and ETS-1 was examined using immunoprecipitation (IP). TPX2 enhanced the accumulation of ETS-1 in the nuclear and the recruitment of its binding element (EST binding site, EBS) located in the promoter region of its downstream gene, *mmp9*. Moreover, TPX2 enhanced the *in vitro* or *in vivo* invasion of a typical endocrine-independent prostate carcinoma cell line, PC-3. Therefore, TPX2 enhanced the activation of the HGF/ETS-1 pathway to enhance the invasion of endocrine-independent prostate carcinoma cells and thus it would be a promising target for prostate carcinoma treatment.

## Introduction

The prognosis for endocrine-independent prostate carcinoma (PC) is still poor due to its insensitivity to androgen deprivation therapy (ADT) or its highly metastatic feature (1). Therefore, it is valuable to explore the detailed mechanisms of resistance and progression in endocrine-independent PC cells, with specific focus on the molecular alterations that participate in the activation of androgen receptor (AR)-independent pathways (2, 3). The PC-3 cell line are a widely used and recognized endocrine-independent PC research model (4). The ETS-1 (E26 transformation specific sequence 1), which belongs to the ETS protein family, is a kind of transcription factor containing the ETS domain (transcription activation domain) and the helix DNA-binding domains (DBD) (5). In the presence or HGF, a typical agonist of the c-MET/ETS-1 pathway, ETS-1 could be translocated from cytoplasm into the nuclear and recruit to the ETS-binding elements (EBS) motif (5’-GGAA/T-3’ sequence) located in the promoter region of the targeted genes (such as matrix metallopeptidases, *mmps*) of ETS-1. Furthermore, the activated ETS-1could promote the invasion of cancerous cells by mediating the transcription of *mmps* (6). Recently, increasing data have confirmed that the high level of ETS-1 in tumor tissues is associated with the prognosis of patients with malignancies, including breast cancer, lung cancer or liver cancer (7). Moreover, the co-factors, especially the co-activators, are essential for the transcription factor activity of ETS-1 (8). Based on the important role of ETS-1in mediating the invasion of cancerous cells, it is valuable to reveal the roles of ETS-1 and its co-factors in endocrine-independent PC cells.

TPX2 (the targeting protein for Xklp2), which is considered as a micro-tubulin interacted protein to promote the proliferation and metastasis of cancerous cells, is featured as containing the TPX domain (9). TPX2 has been considered as an important regulator of human cancers or a promising therapeutic target for treatment, and overexpression of TPX2 is associated with the progress of human malignancies or the poor prognosis of human patients, especially those with PC (10, 11). Recently, increasing data has indicated that TPX2 not only functions by way of modulating tubulin assembling, but could also promote the invasion of cancerous cells *via* some other mechanisms; TPX2 could enhance the metastasis of cancerous cells *via* an enhancement of the expression level of *mmps* (12, 13). In the present study, our results revealed a novel mechanism of TPX2 in its ability to promote the invasion of PC-3 cells, a typical AR-deficiency PC cells, by interacting with ETS-1 and enhancing its transcription factor activation. The results not only elucidated a promising mechanism of TPX2 to mediate the transcription of *mmps*, but also extended our knowledge of endocrine-independent PC.

## Materials and Methods

### Clinical Specimens and Vectors

The clinical specimens diagnosed with endocrine-independent prostate carcinoma were obtained by daily surgical resection with written consent from patients from the period between April 4, 2016 to October 30, 2019. The collection and the usage of clinical specimens was reviewed and approved by the Ethic Committee of the Qujing Affiliated Hospital of Kunming Medical University, Yunnan Province, China. The total mRNA samples were extracted from these clinical specimens and reverse transcribed into cDNA, which were conserved in our lab under the temperature condition of -80°C. The lentivirus particles containing the full-length sequences of TPX2, ETS-1 or the siRNA of NA of TPX2 were purchased from Vigene Corporation, Jinan City, Shandong Province, China. The luciferase reporters (EBS-Luc [ETS-1 binding sites], the EBS [GGAA]_8_ sequences cloned into a pGL4.26 plasmid) were a gift from Dr. Yinjie Gao at the Fifth Medical Center, General Hospital of Chinese PLA (formerly named the Beijing 302^nd^ Hospital of Chinese PLA) (14).

### Cell Cultures and Reagents

The endocrine-independent cell line PC-3 was a gift from Dr. Yinjie Gao at the Fifth Medical Center, General Hospital of Chinese PLA (formerly named the Beijing 302^nd^ Hospital of Chinese PLA) (14). The recombinant human HGF (hepatocyte growth factor) was purchased from Pepro-Tech Corporation (Rocky Hill, NJ, USA). The HEK293 or the PC-3 cells were cultured in high-sugar complete Dulbecco’s modified Eagle medium (DMEM, Invitrogen, Carlsbad, CA, UAS) with 10% fetal bovine serum (FBS, Hyclone, Thermo Fisher Scientific, Waltham, MA, USA) and cultured at 37°C, 5% CO_2_ condition.

### Luciferase Assay

PC-3 cells which were transfected with plasmids were seeded in the 24-well plates (Corning, Corning, NY, USA) containing phenol red-free DMEM (Dulbecco’s Modified Eagle’s medium) (Invitrogen, Carlsbad, CA, USA) and 0.5% charcoal-stripped FBS (fetal bovine serum) (Hyclone, Thermo Fisher Scientific, Waltham, MA, USA) was added with 10ng/L HGF or without HGF (the solvent control). The luciferase activation or the activation of β-galactosidase (used as the loading control) were examined using a kit purchased from Promega Corporation (Madison, WI, USA), according to the instructions provided by the manufacturer or the methods described by Feng et al. (15).

### qPCR Analysis

The total RNA samples were extracted from the PC-3 cells or the prostate clinical specimens using a PARISTM Kit (Thermo Fisher Scientific, Waltham, MA, USA) and the RNA samples were reverse transcribed into cDNA using a Multiscribe™ Reverse Transcriptase kit (Thermo Fisher Scientific, Waltham, MA, USA). Next, a quantitative polymerase chain reaction (qPCR) was performed according to the instructions from the manufacturer and methods described in the previous publication (16, 17). The level of β-actin mRNA was measured as a loading control. The primers used in qPCR experiments are listed as: (1) *mmp3* Forward sequence: 5’-CACTCACAGACCTGACTCGGTT-3’, *mmp3* Reverse sequence: 5’-AAGCAGGATCACAGTTGGCTGG-3’; (2) *β-actin* Forward sequence 5’-CACCATTGGCAATGAGCGGTTC-3’; *β-actin* Reverse sequence:5’-AGGTCTTTGCGGATGTCCACGT-3’; (3) *tpx2* forward sequence: 5′-ACCTTGCCCTACTAAGATT-3′; *tpx2* reverse: 5′-AATGTGGCACAGGTTGAGC-3′; (4) *mmp9* Forward sequence: 5-GCCACTACTGTGCCTTTGAGTC-3’; *mmp9* Reverse sequence 5’-CCCTCAGAGAATCGCCAGTACT-3’.

### Western Blot Experiments and Cellular Sub-Fraction Analysis

The PC-3 cells, which were transfected with plasmids or treated with 10ng/ml concentration of HGF, were harvested for western blot experiments, which were performed using a standard protocol and described by Wang et al. and Ma et al. (18, 19). The protein level of TPX2, ETS-1, MMP3 or MMP9 was examined by their antibodies (Abcam Corporation, Cambridge, CB2 0AX, UK). The GAPDH was used as a loading control. The western blot images were quantitatively examined using Image J software (National Institutes of Health [NIH], Bethesda, MD, USA) (20). A cellular sub-fraction analysis was performed in order to examine the subcellular accumulation of TPX2 or ETS-1 in PC-3 cells. The PC-3 cells, which were transfected with plasmids or treated with HGF, were harvested for subcellular sub-fraction experiments, which were performed following the methods described by Yang et al. or Zhang et al. (21, 22). Lamin A was used as the indicator of the nuclear sub-fraction, whereas β-actin was chosen as the indicator for the cytoplasm sub-fraction of the PC-3 cells. The expression level of ETS-1, PTX2, Lamin A or β-actin was detected by their antibodies (Abcam Corporation, Cambridge, CB2 0AX, UK).

### Chromatin Immunoprecipitation (ChIP) Experiments

The ARQ-197, Paclitaxel or the Vincristine was purchased from the Selleck Corporation, Huston, Texas, USA. After PC-3 cells were transfected with the corresponding vector, firstly pre-treat PC-3 cells with Paclitaxel or the Vincristine at a dose of 10 nmol/L or ARQ-197 at a dose of 3 μmol/L for 2-4 hours, and then treat the cells with HGF at a dose of 10 ng/ml about 30min-40min. After the treatment, cells were harvested for the Chromatin immunoprecipitation (ChIP) experiments. The ChIP was performed following the instructions provided by the manufacturer (the ChIP kit, Upstate Corporation, Richfield Springs, NY, USA) and the methods described in previous publications (23). The complex between DNA and ETS-1 was separated from the PC-3 cells by using co-immunoprecipitation (co-IP) *via* ETS-1’s antibody. The recruitment of ETS-1 to the promoter region of its downstream gene (*mmp9*) was examined by the qPCR. The primers used in the ChIP experiments were listed as: MMP9 promoter forward: 5′- TACATTGGTACCTCTTGGGTCTTGGCCTTAGT -3′; MMP9 promoter reverse: 5′- TTGATACTCGAGCCAGCACCAGGAGCACC -3′.

### The Transwell Experiment

The invasion (invasive growth) of PC-3 cells was examined using the invasion-transwell analysis (24). PC-3cells, which were transfected with plasmids, were seeded into the transwell plates (24‐well plates with transwell chambers) (Cat. No.: Costar 3422, Corning, Corning, NY, USA) fitted with a polyethylene terephthalate filter membrane with 8‐μm pores. The membrane undersurface of the chambers was pre-covered with ECM gel (extracellular matrix gel) from Engelbreth‐Holm‐Swarm mouse sarcoma (BD Biosciences) and the top surface of the transwell chambers were filled with cell-suspension (5 × 105 cells/ml) in serum‐free medium, and the bottoms were filled with medium containing 10% FBS (17). The invaded cells were fixed and stained with crystal violet (0.25 w/v), and the images of the wells were quantitatively analyzed using Image J software.

### The *In Vivo* Invasion of PC-3 Cells in Nude Mice

All the methods or protocols and the usage of animals were reviewed and approved by the ethics committee of the Qujing Affiliated Hospital of Kunming Medical University, Yunnan Province, China. All studies were carried out in accordance with the UK Animals (Scientific Procedures) Act 1986 and the associated guidelines. The nude mice were purchased from the Si-Bei-Fu Corporation, Beijing, China and were 4–6 weeks’ old. PC-3 cells were mixed with the medical hydrogel to form the hydrogel-drips (5000 cells per drip), which were adhered onto the surface of the liver organs of nude mice following the methods describes by Wei et al. and Ma et al. (25, 26). The mice received micro-PET screening and the liver organs with nodules formed using PC-3 cells were harvested, and the invasion depth (the PC-3 cells were firstly adhered to the surface of the liver organs and broke the surface to invade the liver tissues) of the PC-3 cells were examined by H&E staining (25, 26). The images of H&E staining were quantitatively analyzed using Image J software (25, 26).

### Statistical Analysis

Statistical analysis was performed by Bonferroni correction, with or without a two-way analysis of variance using SPSS Software (Version No.: 6.0; IBM Corporation, Armonk, NY, USA). A P-value of <0.05 was considered statistically significant.

## Results

### TPX2 Enhanced the Transcription Factor Activation of ETS-1

**Figure 1:**
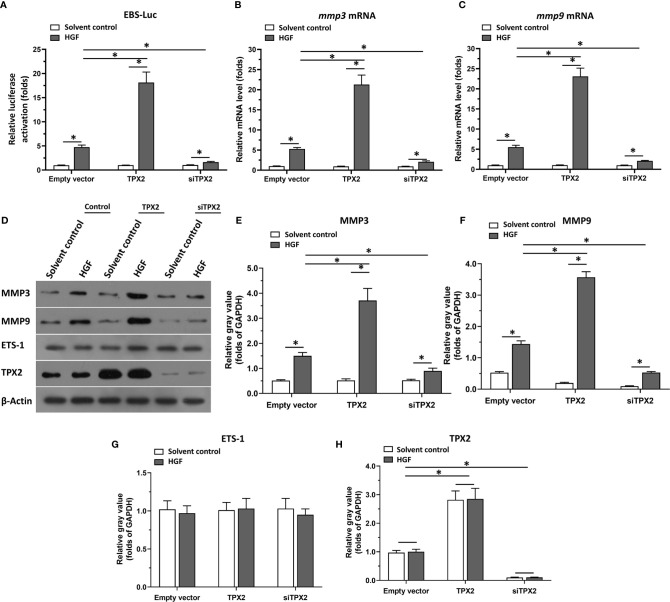
TPX2 enhanced the transcription factor activation of ETS-1. Cells were treated with the solvent control or 10ng/ml concentration of HGF. PC-3 cells which were co-transfected with luciferase reporter (EBS-Luc) and the control, TPX2 or siTPX2, were harvested for luciferase analysis **(A)**. **(B, C)** PC-3 cells which were transfected with the control, TPX2 or siTPX2, were harvested for qPCR to examine the mRNA level of *mmp3* **(B)** or *mmp9* **(C)**. **(D–H)** The expression of TPX2, MMP3 or MMP9 in PC-3 cells were examined by western blot. The results were shown as western blot images **(D)** or the quantitative results of the western blot **(E–H)**. *P<0.05.

Firstly, the effect of TPX2 on ETS-1’s transcription factor activation was examined by the luciferase-activation examination or the qPCR methods. As shown in **Figure 1A**, overexpression of TPX2 enhanced the activation of ETS-1 induced by HGF, whereas knockdown of TPX2 *via* its siRNA decreased the activation of ETS-1 induced by HGF. To further examine the effect of TPX2 on EST-1, the mRNA or protein level of ETS-1’s downstream genes, *mmp3* or *mmp9*, was examined. As shown in **Figures 1B, C**, overexpression of TPX2 enhanced the mRNA level of *mmp3* or *mmp9* induced by HGF. Knockdown of TPX2 decreased the mRNA level of *mmp3* and *mmp9* (**Figures 1B, C**). Similar results were obtained from the western blot which examined the protein level of MMP3 or MMP9 (**Figures 1D–H**). Therefore, TPX2 enhanced the transcription factor activation of ETS-1.

### TPX2 Interacted With ETS-1

**Figure 2:**
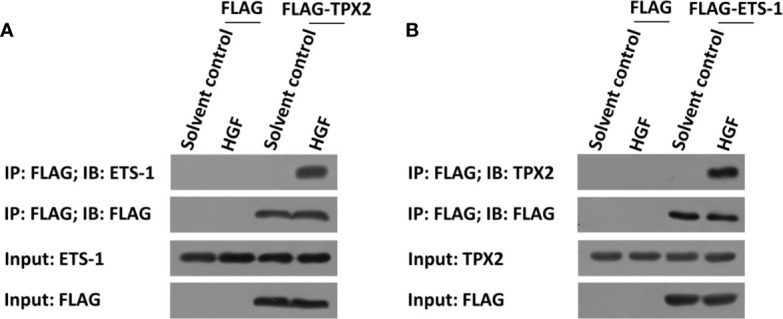
TPX2 interacted with ETS-1 in PC-3 cells. Cells were treated with the solvent control or 10ng/ml concentration of HGF. **(A)** PC-3 cells were transfected with FLAG or FLAG-TPX2. **(B)** PC-3 cells were transfected with FLAG or FLAG-ETS-1. The cells were analyzed using the IP methods.

The above data indicated that TPX2 enhanced the transcription factor activation of ETS-1. To further examine the effect of TPX2, the interaction between TPX2 and ETS-1 was examined in PC-3 cells. As shown in **Figure 2**, FLAG-TPX2 or FLAG-ETS1 could interact with ETS1 or TPX2 in PC-3 cells. Therefore, TPX2 would modulate the activation of ETS-1 *via* the protein-protein interaction.

### TPX2 Enhanced the Accumulation of ETS-1 in the Nuclear and the Recruitment of ETS-1 to Its Downstream Gene Promoter Region

**Figure 3:**
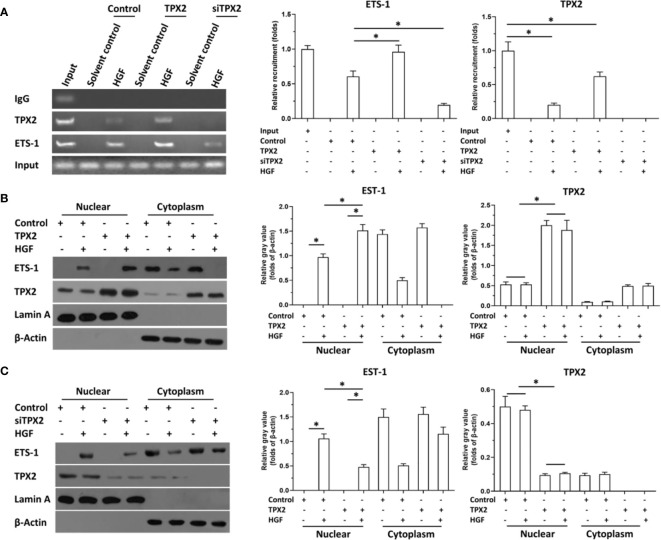
TPX2 enhanced the recruitment of ETS-1 to *mmp9*’s promoter or the accumulation of ETS-1 in PC-3 cells. The cells were treated with the solvent control or 10ng/ml concentration of HGF. PC-3 cells were transfected with the control, TPX2 or siTPX2. **(A)** cells were harvested for ChIP to examine the recruitment of ETS-1 to *mmp9*’s promoter region. **(B, C)** cells were harvested for cellular sub-fraction to examine the accumulation of ETS-1 or TPX2 in the sub-fraction of PC-3 cells. The results were shown as images or the quantitative results were shown. *P<0.05.

Then, the effect of TPX2 on the accumulation of ETS-1 in the nuclear and the recruitment of ETS-1 to its downstream gene promoter region was examined using cellular sub-fraction analysis or the ChIP experiments. As shown in **Figure 3A**, HGF induced the recruitment of ETS-1 to *mmp9*’s promoter. Overexpression of TPX2 enhanced the recruitment of ETS-1 to its downstream gene promoter region induced by the HGF treatment, whereas knock down of TPX2 decreased the recruitment of ETS-1 to its downstream gene’s promoter region induced by the HGF treatment. Moreover, as shown in **Figures 3B, C**, HGF could induce the accumulation of ETS-1 in PC-3 cells’ nuclear. Overexpression of TPX2 enhanced the accumulation of ETS-1 in the nuclear induced by the HGF treatment, whereas knock down of TPX2 decreased the accumulation of ETS-1 in the nuclear induced by the HGF. Therefore, TPX2 enhanced the accumulation of ETS-1 in the nuclear and the recruitment of ETS-1 to its downstream gene promoter region to enhance the transcription factor activation of ETS-1.

### The Expression of *ets-1*, *tpx2* or *mmps* in Clinical Specimens

**Figure 4:**
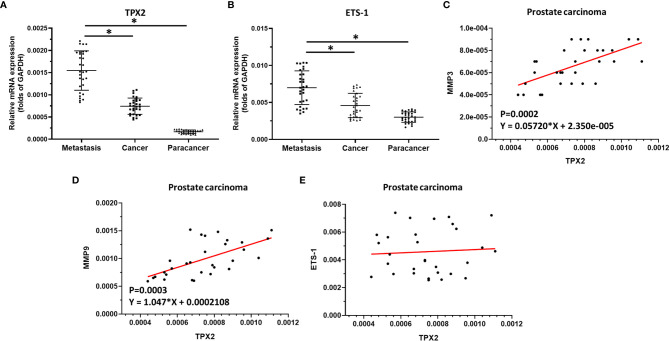
The significance of TPX2 in clinical specimens. **(A)** The expression of *tpx2* in non-tumor specimens, primary prostate carcinoma specimens or metastatic prostate specimens by qPCR. **(B)** The expression of *ets-1* in non-tumor specimens, primary prostate carcinoma specimens or metastatic prostate specimens by qPCR. **(C–E)** The co-relationship between *tpx2* and *mmp3* **(C)** or mmp9 **(D)** or *ets-1* **(E)** was shown as scatter-plot images. *refers multiplication sign.

The above result revealed the effect of TPX2 on ETS-1’s activation. To confirm the significance of the TPX2-ETS-1 axis, the expression of *ets-1, tpx2 or mmps* in clinical specimens was examined by qPCR. As shown in **Figure 4**, the mRNA expression level of *tpx2* or *ets-1* was much higher in prostate carcinoma than it was in prostate non-tumor tissues (**Figures 4A, B**). Moreover, the expression of *tpx2* or *ets-1* is much higher in the metastatic prostate carcinoma than in primary prostate carcinoma (**Figures 4A, B**). Next, the correlation between *tpx2* with *mmp3*, *mmp9* or *ets-1* in prostate carcinoma was examined. As shown in **Figures 4C–E**, the expression of *tpx2* was positively correlated with the expression level of *mmp3* (P=0.0002; Y = 0.05720*X + 2.350e-005) (**Figure 4B**) or *mmp9* (P=0.0003; Y = 1.047*X + 0.0002108) (**Figure 4C**); whereas the mRNA of *tpx2* was not correlated with *ets-1*. These results further confirmed the effect of TPX2 on ETS-1’s activation.

### The Specificity of TPX2 on ETS-1

Firstly, the involvement of microtubules in TPX2 on ETS-1 was examined. The microtubule depolymerization inhibitor Paclitaxel and the microtubule polymerization inhibitor Vincristine were used. The results showed that Paclitaxel enhanced the effect of TPX2 on ETS-1’s recruitment to *mmp9*’s promoter region, while Vincristine can down-regulate the effect of TPX2 on ETS-1 (**Supplemental Figure 1**). To further examine the effect of TPX2 on HGF/ETS-1 pathway, ARQ-197, the inhibitor of HGF/ETS-1 pathway, was used. As shown in **Supplemental Figure 2**, ARQ-197 almost blocked the activation of luciferase reporter, EBS-Luc, induced by HGF, in the presence of TPX2. Therefore, TPX2 may affect ETS-1 through the microtubule system and the activation of ETS-1 is dependent on its upstream pathway.

### TPX2 Enhanced the *In Vitro* and *In Vivo* Invasion of PC-3 Cells

**Figure 5:**
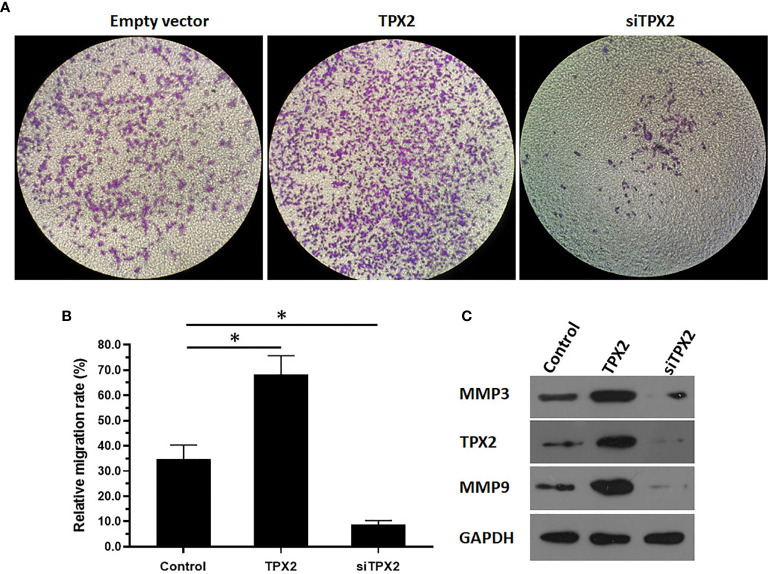
TPX2 enhanced the in vitro invasion of PC-3 cells. PC-3 cells were transfected with the control, TPX2 or siTPX2. The cells were harvested for the transwell experiments. The results were shown as images of the invaded cells **(A)** or the quantitative results of the images **(B)** were shown. The protein level of TPX2, MMP3 or MMP9 in PC-3 cells was examined by western blot **(C)**. *P<0.05.

Because ETS-1 is a transcription factor that functions as a primary regulator in the metastasis process by mediating the expression of MMP to degrade the ECM of cancer cells and promote the invasion or migration of cancer cells, the effect of TPX2 on the invasion of PC-3 cells was examined. As shown in **Figure 5**, the results from the transwell experiments indicated that overexpression of TPX2 enhanced the *in vitro* invasion of PC-3 cells, whereas knockdown of TPX2 suppressed the *in vitro* invasion of PC-3 cells.

**Figure 6:**
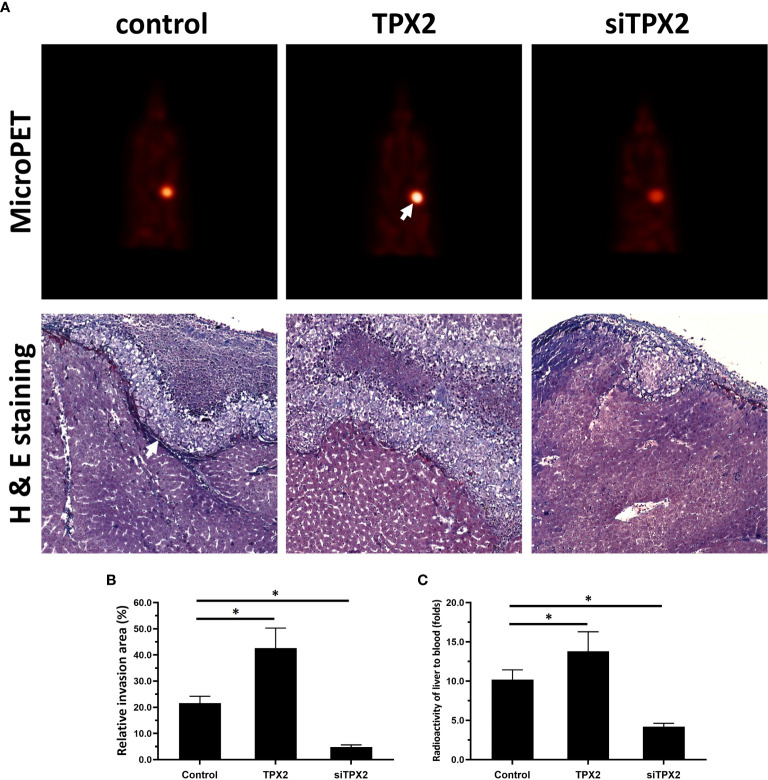
TPX2 enhanced the in vivo invasion of PC-3 cells. PC-3 cells were transfected with the control, TPX2 or siTPX2. The cells were mixed with hydrogel and adhered onto the surface of the liver organs of nude mice. The lesions formed by the PC-3 cells were measured using micro-PET and pathological analysis. The results were shown as micro-PET images or pathological analysis **(A)** and the quantitative results of the images **(B, C)**. *P<0.05.

Moreover, the *in vivo* growth of PC-3 cells was examined. As shown in **Figure 6**, overexpression of TPX2 promoted the subcutaneous growth of PC-3 cells, whereas knockdown of TPX2 repressed the subcutaneous growth of PC-3 cells. The subcutaneous tumor model is a common model; however, this model could not reflect the *in vivo* invasion of cells. Therefore, the *in vivo* invasion of PC-3 cells was performed in the liver organs of nude mice. As shown in **Figure 6**, the intrahepatic growth of PC-3 cells in the liver organs of nude mice was revealed using micro-PET images. Moreover, the results of the H&E staining revealed that PC-3 could invade from the surface of the liver organ into the liver tissue to form nodules in the nude mice’s liver organs. Overexpression of TPX2 promoted the intrahepatic invasion of PC-3 cells, whereas knockdown of TPX-2 repressed the intrahepatic invasion of PC-3 cells in the liver organs of nude mice. Therefore, TPX2 enhanced the *in vitro* and *in vivo* invasion of PC-3 cells.

## Discussion

TPX2 is a tubulin-associated protein considered as a proto-oncogene in human cancer cells. A high level of TPX2 has been detected in several kinds of human cancer, such as bladder cancer, breast cancer, HCC, and especially prostate cancer (9, 10). Previous research has focused on the role of TPX2 in centrosome formation and cell mitosis, and TPX2 is closely related to microtubules (11, 12). The microtubule system is not only the basis for maintaining the morphology of cells but is also key to the transport of materials within cells (27). Thus, TPX2 might regulate the activation of important transcription factors by modulating their translocation from cytoplasm to nucleus. In the present work, the protein interaction between TPX2 and ETS-1 was identified by using co-IP methods. Overexpression of TPX2 enhanced the activation of the ETS-1 pathway and enhanced the invasion of PC-3 cells by enhancing the expression of ETS-1’s invasion-related downstream genes, i.e. *mmp3* or *mmp9*. The mechanisms data indicated that TPX2 could modulate the activation of the ETS-1 pathway by promoting the accumulation of ETS-1 in the nuclear and the recruitment of ETS-1 to its downstream gene promoter region. Microtubule was involved in the effect of TPX2 on ETS-1. Paclitaxel can inhibit the depolymerization of the microtubule system and strengthen the microtubules in the cell; vincristine can inhibit the polymerization of microtubules and destroy the microtubule structure in the cell. The results show that paclitaxel can up-regulate the effect of TPX2, while vincristine can inhibit the effect of TPX2. Therefore, the results are of great significance; the regulation of ETS-1 activity depends not only on its activation by upstream signaling pathways such as HGF, but also on co-factors such as SRC (steroid receptor coactivator), and by microtubule-related mechanisms. Among these factors, HGF acts on the upstream signaling pathway to activate it, activated ETS-1 migrates from the cytoplasm to the nucleus (28) and the co-factors modulate the recruitment of ETS-1 to promoter regions (29, 30). During this process, the nuclear-plasmid migration of ETS-1 is essential for its activation. Our results indicated that TPX2 promoted the cytoplasmic migration of ETS-1, which not only expands our understanding of the regulation mechanism of transcription factor activity, but also deepens our understanding of the cytoskeleton affecting the activity of cell signaling pathways. Some previous data showed that TPX2 could promote the invasion of cancerous cells by enhancing the expression of MMPs and our data provided a promising mechanism of TPX2’s effect on MMPs (12, 13).

The highly aggressive feature of cancerous cells is reflected in the invasive growth of malignant cells that gradually destroy the normal structure of tissues in the primary location and are translocated to another location (the metastatic process) (31, 32). During disease progression, tumor cells can break through the restrictions of the extracellular matrix and migrate to other tissues and organs (31, 32). In these processes, the destruction of the local extracellular matrix by tumor cells and the destruction of the extracellular matrix at the site of metastasis during metastasis are the key steps for its proliferation, metastasis, and invasion (33–35). This step is mediated by ETS -1; and the activated ETS-1 can destroy the extracellular matrix and normal tissue structure by mediating its downstream genes (the various matrix metalloproteinases) (36–38). In the present work, the invasion of PC-3 cells was examined using the *in vitro* (transwell) or *in vivo* model (the nude mice model). In the nude mice model, PC-3 cells were adhered to the surface of liver organs by using a hydrogel droplet containing PC-3 cell. The cells broke through the liver surface and invaded the liver organs to form the tumor lesions/nodules. The invasion of PC-3 cells can be quantitatively studied by the depth of cell invasion. The intrahepatic invasion model used in this study was originally intended to quantitatively reflect the invasion ability of PC cells in solid organs (such as liver) based on the depth of Lesions/nodules invasion in liver organs. The main results of this study were that TPX2 up-regulates the expression of MMPs by interacting with ETS-1, and the expression level of MMPs is closely related to the invasion ability of cells, so the application of intrahepatic invasion model in this study is appropriate. At the same time, liver metastases from prostate cancer have also been reported, and the results of this study are therefore helpful for clinical diagnosis and treatment of prostate cancer metastasis. Bone metastasis is the most common type of PC metastasis. The invasion of prostate cancer cells on other organs such as liver, lung, kidney and adrenal glands has also been reported (39).

Moreover, although the prognosis of PC has improved in recent years, a large number of PC patients are not responsive enough to androgen deprivation therapy (ADT) and these patients are often considered as endocrine-independent PC. Many mechanisms have been used to explore ADT-resistance and the progression of PC: (1) the high frequency of AR aberrations were often identified in ADT-resistance PC patients suggesting that AR-mutation is one of the main drivers of progression and the poor prognosis of PC patients (40); (2) some poorly differentiated or highly aggressive PC cells or specimens show low levels of AR or PSA (41); (3) some AR-independent pathways function as an alternative mechanism contributing to PC and sustain the proliferation or invasion in a completely hormone-independent manner (42); and (4) some factors or pathways mediating the stemness signatures or self-renewal often participate in the recurrence or ADT-resistance of PC (43). Therefore, it is valuable to explore these detailed mechanisms and the therapeutic strategies of endocrine-independent (androgen receptor [AR]-independent) PC in order to improve the survival rate of patients. In the present work, our results revealed the role of TPX2/ETS-1 interaction in promoting the invasion of AR-independent PC cells and indicated that TPX2/ETS-1 could be a promising target for PC treatment. Our results not only expand on the understanding of ETS-1 and TPX2 in PC, but also help provide more options for PC treatment.

Recently, results of a clinical trial (the COSMIC-021 trial) mentioned that Cabozantinib (an anti-angiogenesis drug that also targets MET) could be benefit for treatment (44). Therefore, it is valuable to focus on the significance of c-MET and the small molecular inhibitor of c-MET in the PC treatment (45, 46). Another report revealed that the HGF/ETS pathway could be active in the TCGA molecular subgroup of TMPRSS2/ERG fused prostate cancers (50%) (47). The activation of HGF/ETS pathway is regulated by many factors, such as the expression level of c-MET and the expression level of co-regulatory factors of ETS-1. Therefore, it is necessary to detect the expression levels of related factors in PCs of different pathological subtypes in the future.

## Conclusions

In summary, in the present work, the results indicated that TPX2 promotes the invasion of endocrine-independent prostate carcinoma *via* its interaction with ETS-1.

## Data Availability Statement

The raw data supporting the conclusions of this article will be made available by the authors, without undue reservation.

## Ethics Statement

The animal study was reviewed and approved by Ethic Committee of the Qujing Affiliated Hospital of Kunming Medical University, Yunnan Province, China.

## Author Contributions

All authors contributed to the article and approved the submitted version.

## Funding

Financial support for this study was supported by Yunnan Fundamental Research Projects (grant NO. 2019FE001 [-277]), Yunnan health training project of high level talents and Qujing Affiliated Hospital of Kunming Medical University (2019YJKT11 and 2020YJKT03).

## Conflict of Interest

The authors declare that the research was conducted in the absence of any commercial or financial relationships that could be construed as a potential conflict of interest.

## Supplementary Material

Supplementary Figure 1 The involvement of microtubule in the effect of TPX2 on ETS-1. After PC-3 cells were transfected with the corresponding vector, firstly pre-treat PC-3 cells with Paclitaxel or the Vincristine at a dose of 10 nmol/L for 2-4 hours, and then treat the cells with HGF at a dose of 10 ng/ml about 30min-40min. After the treatment, cells were harvested for the Chromatin immunoprecipitation (ChIP) experiments. The results were shown as the histogram of mean ± SD from qPCR. *P<0.05.

Supplementary Figure 2 The specificity of TPX2 on HGF/ETS-1 pathway by ARQ1-97. After PC-3 cells were transfected with the corresponding vector, firstly pre-treat PC-3 cells with ARQ-197 at a dose of 3μmol/L for 2-4 hours, and then treat the cells with HGF at a dose of 10 ng/ml about 24h. After the treatment, cells were harvested for the luciferase. The results were shown as the histogram of mean ± SD from luciferase. *P<0.05.
