# Corrigendum: TPX2 enhanced the activation of the HGF/ETS-1 pathway and increased the invasion of endocrine-independent prostate carcinoma cells

**DOI:** 10.3389/fonc.2022.1032265

**Published:** 2022-09-22

**Authors:** Hongqing Zhou, Mingsheng Liu, Tao Shao, Pingbo Xie, Shaojie Zhu, Wei Wang, Qiong Miao, Jiaxi Peng, Peng Zhang

**Affiliations:** ^1^ The Second Ward of Urology, Qujing Affiliated Hospital of Kunming Medical University, Qujing, China; ^2^ Department of Urology, Chinese People’s Liberation Army (PLA) General Hospital/Chinese PLA Medical Academy, Beijing, China

**Keywords:** the targeting protein for Xklp2, prostate carcinoma, E26 transformation specific sequence 1, metastasis, endocrine-independent

An author name was incorrectly spelled as Qinghong Zhou. The correct spelling is Hongqing Zhou.

The authors apologize for this error and state that this does not change the scientific conclusions of the article in any way. The original article has been updated.

## Publisher’s note

All claims expressed in this article are solely those of the authors and do not necessarily represent those of their affiliated organizations, or those of the publisher, the editors and the reviewers. Any product that may be evaluated in this article, or claim that may be made by its manufacturer, is not guaranteed or endorsed by the publisher.

