# Supplementary material for: TPX2 Enhanced the Activation of the HGF/ETS-1 Pathway and Increased the Invasion of Endocrine-Independent Prostate Carcinoma Cells
Source: Front Oncol. 2021 May 28;11:618540. doi: 10.3389/fonc.2021.618540 (PMC8193931; doi:10.3389/fonc.2021.618540)
Supplement: Supplementary Figure 1 — The involvement of microtubule in the effect of TPX2 on ETS-1. After PC-3 cells were transfected with the corresponding vector, firstly pre-treat PC-3 cells with Paclitaxel or the Vincristine at a dose of 10 nmol/L for 2-4 hours, and then treat the cells with HGF at a dose of 10 ng/ml about 30min-40min. After the treatment, cells were harvested for the Chromatin immunoprecipitation (ChIP) experiments. The results were shown as the histogram of mean ± SD from qPCR. *P<0.05. [file DataSheet_1.doc]

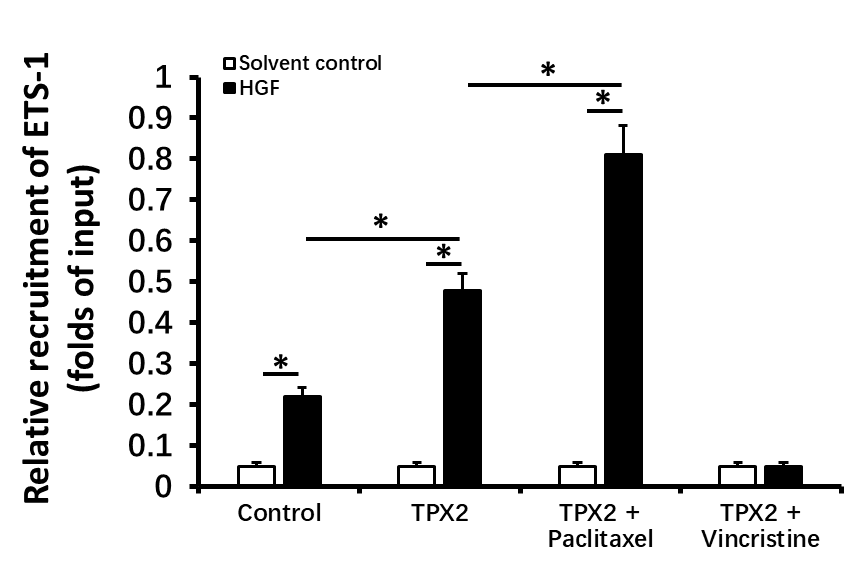


Supplemental Figure 1. The involvement of microtubule in the effect of TPX2 on ETS-1. After PC-3 cells were transfected with the corresponding vector, firstly pre-treat PC-3 cells with Paclitaxel or the Vincristine at a dose of 10 nmol/L for 2-4 hours, and then treat the cells with HGF at a dose of 10 ng/ml about 30min-40min. After the treatment, cells were harvested for the Chromatin immunoprecipitation (ChIP) experiments. The results were shown as the histogram of mean ± SD from qPCR. *P<0.05
