# Supplementary material for: TPX2 Enhanced the Activation of the HGF/ETS-1 Pathway and Increased the Invasion of Endocrine-Independent Prostate Carcinoma Cells
Source: Front Oncol. 2021 May 28;11:618540. doi: 10.3389/fonc.2021.618540 (PMC8193931; doi:10.3389/fonc.2021.618540)
Supplement: Supplementary Figure 2 — The specificity of TPX2 on HGF/ETS-1 pathway by ARQ1-97. After PC-3 cells were transfected with the corresponding vector, firstly pre-treat PC-3 cells with ARQ-197 at a dose of 3μmol/L for 2-4 hours, and then treat the cells with HGF at a dose of 10 ng/ml about 24h. After the treatment, cells were harvested for the luciferase. The results were shown as the histogram of mean ± SD from luciferase. *P<0.05. [file DataSheet_2.doc]

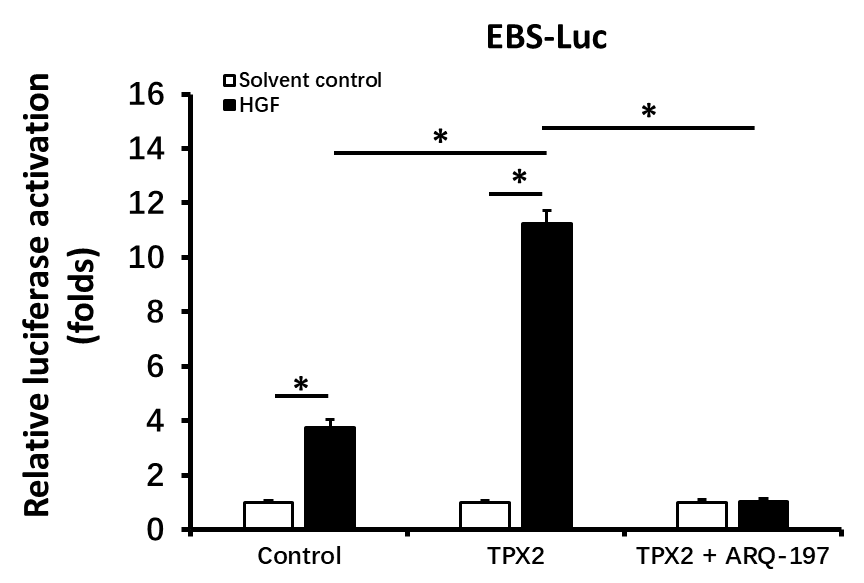


Supplemental Figure 2. The specificity of TPX2 on HGF/ETS-1 pathway by ARQ1-97. After PC-3 cells were transfected with the corresponding vector, firstly pre-treat PC-3 cells with ARQ-197 at a dose of 3μmol/L for 2-4 hours, and then treat the cells with HGF at a dose of 10 ng/ml about 24h. After the treatment, cells were harvested for the luciferase. The results were shown as the histogram of mean ± SD from luciferase. *P<0.05
